# Advances in Laser Additive Manufacturing of Cobalt–Chromium Alloy Multi-Layer Mesoscopic Analytical Modelling with Experimental Correlations: From Micro-Dendrite Grains to Bulk Objects

**DOI:** 10.3390/nano12050802

**Published:** 2022-02-26

**Authors:** Muhammad Arif Mahmood, Asif Ur Rehman, Carmen Ristoscu, Mehmet Demir, Gianina Popescu-Pelin, Fatih Pitir, Metin Uymaz Salamci, Ion N. Mihailescu

**Affiliations:** 1 Mechanical Engineering Program, Texas A&M University at Qatar, Doha P.O. Box 23874, Qatar; muhammad.arif_mahmood@outlook.com; 2 Laser Department, National Institute for Laser, Plasma and Radiation Physics (INFLPR), 077125 Magurele, Ilfov, Romania; gianina.popescu@inflpr.ro (G.P.-P.); ion.mihailescu@inflpr.ro (I.N.M.); 3 ERMAKSAN, Bursa 16065, Turkey; mehmetdemir4994@gmail.com (M.D.); fatih.pitir@ermaksan.com.tr (F.P.); 4 Department of Mechanical Engineering, Gazi University, Ankara 06570, Turkey; msalamci@gazi.edu.tr; 5 Additive Manufacturing Technologies Research and Application Center-EKTAM, Gazi University, Ankara 06560, Turkey

**Keywords:** laser powder bed fusion, Cobalt–chromium alloy, dendrite grain size, hardness, mesoscopic analytical modelling

## Abstract

This study presents two analytical models for the laser powder bed fusion (LPBF) process. To begin, the single layer’s dimensions were measured using principal operating conditions, including laser power, laser scanning speed, powder layer thickness, and hatch distance. The single-layer printing dimensions were transformed into multi-layer printing using the hatch distance. The thermal history of the printed layers was used as an input to the Johnson–Mehl–Avrami-Kolmogorov model to estimate the average dendrite grain size. LPBF experiments were conducted for a Cobalt–chromium (Co–Cr) alloy to validate the developed model. The average dendrite grain size was estimated using a scanning electron microscope (SEM) combined with “Image J” software. The Vickers hardness test was performed to correlate the average dendrite grain size and operating conditions. A 10–15% mean absolute deviation was presented between experiments and simulation results. In all samples, a Co-based γ-FCC structure was identified. An inverse correlation was established between the laser power and smaller average dendrite grain, while a direct relationship has been determined between laser scanning speed and average dendrite grain size. A similar trend was identified between hatch distance and average dendrite grain size. A direct link has been determined between the average dendrite grain size and hardness value. Furthermore, a direct relationship has connected the laser volume energy density and hardness value. This study will help experimentalists to design operating conditions based on the required grain size and corresponding mechanical characteristics.

## 1. Introduction

The additive manufacturing (AM) process can produce three-dimensional (3D) parts, by utilizing layer-upon-layer technique, via a computer-aided design (CAD) model [1]. AM has been successfully applied in medical [2,3], aerospace [4], automotive [5], and various industrial applications [6]. The Hall–Petch relationship identifies that the material’s active strength depends on the grain size such that strength elevated by decreasing the average grain size [7]. From literature, it has been identified that the developed grains are characterized via average size, developed phases, and positioning [8]. Average grain sizes can be measured using experimental and simulation techniques.

Various experiments have been carried out to correlate the operating conditions with the average grain size. Laser additive manufacturing (LAM) techniques can be classified into laser powder bed fusion (LPBF) and laser melting deposition (LMD) [9,10,11]. The rate of heating and solidification play an important role on the final average grain size grain size [12,13,14]. The shape, microstructure, and mechanical properties of LAM-produced 316L stainless steel specimens were investigated [15,16]. It was found that the operating parameters, especially the heat input, play an important role in defining the manufactured part’s grain size. The authors identified that the deposited grains contained austenite grains. Recently, an infrared red camera was applied to monitor the grain evolution in the case of AISI 316L stainless steel based on the heating and cooling rates involved in LAM [17]. It was identified that the cooling rate controls the size of developed grain. In another study, experiments were performed to develop a correlation between number of the deposited layers and average grain size [18]. It was found that with an increase in number of layers, the material experiences thermal cyclic loading, leading to reduced grain size. An investigation was carried out to identify the significant parameters to in the case of titanium alloy-AM [19]. For this purpose, a response surface approach was applied, which assisted in defining optimum parameters. 

For LAM processes, the finite element (FE) and analytical simulation software systems have recently been introduced to analyze the grain size. Recently, the heat-transfer model was linked with the cellular automata (CA) model to simulate the grain formation [20]. In another study, a 3D heat distribution model was linked with 3D CA model to predict the 3D grain evolution [21,22,23]. Besides FE simulations, analytical models were also developed by linking the heat distribution model with the Johnson–Mehl–Avrami–Kolmogorov (JMAK) model in combination with heating and cooling rates of the part [24]. All the developed models were able to estimate results with a reliable accuracy.

In this study, efforts have been carried out to establish a correlation among primary operating conditions, average grains size, and hardness value. For multi-layers printing via the LPBF process, an analytical model has been developed to estimate the dimensions of the deposited layers and corresponding average size and hardness. For this purpose, an analytical solution was initially inferred to calculate the single layer’s dimensions, including height, width, and depth. The single-layer printing was converted into multi-layers printing using a hatch distance. The developed thermal history was used in correlation with the JMAK model [25] to calculate average grain size in the case of multiple layers. To verify the model for multi-layers printing, experiments were carried out for the LPBF of Co–Cr alloy. The average grain size was counted using a scanning electron microscope (SEM). To develop a correlation between operating conditions and hardness value, Vickers hardness testing was carried out.

## 2. Mathematical Modelling

**Figure 1:**
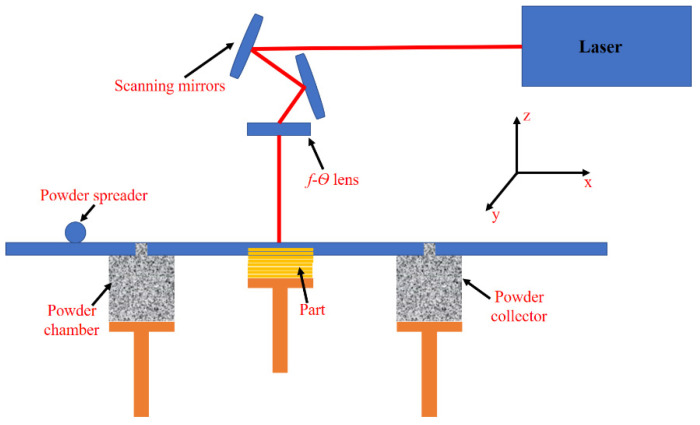
Schematic of the laser powder bed fusion process.

Laser powder bed fusion (LPBF) is an additive manufacturing method developed to liquefy and fuse powder particles via a laser beam. Figure 1 shows the schematic of the LPBF process. Using a mounted piston, the substrate moves downward, giving a space of single-layer thickness. The powder is then uniformly distributed by a powder re-coater (distributor). The laser beam melts the powder and deposits the first layer, which is regulated and guided by *x*-*y* scanning mirrors and *f*-*θ* lens. Following that, the substrate descends to deposit the subsequent layer. The 3D component, designed using computer-aided design/computer-aided manufacturing (CAD/CAM) software, is manufactured by repeating the abovementioned steps [26].

To develop analytical relations for the dimensions of the printed layer, the following assumptions have been taken into account:

**Assumptions** **1.** *The speed of the laser beam (V_s_) is constant, and the focused laser spot is circular. The printed layer’s shape is taken as elliptical and surface tension has been neglected. The thickness (h) of the deposited layer is known.*

**Assumptions** **2.** *The laser beam energy dissipation by the powder granules is constant, and the powder particles’ mean size is taken into consideration [27,28]. While forming a powder bed, the overlapping by the powder particles is ignored. The laser beam absorption and reflection are considered by a cumulative factor, laser absorption coefficient* (α).

**Assumptions** **3.** *To deposit the n-number of layers, the primary operating conditions such as laser power, laser scanning speed, hatch distance, and powder layer thickness were kept constant.*

**Assumptions** **4.** The overlapping (OL) of deposited layers is a hatch distance (H) function. For simplicity, the OL between two layers has been represented by an Equilateral triangle having a height ( $Z^{*}$), designated as OL intersection.

### 2.1. Single Layer Printing

In the LPBF process, the printed layer’s width is usually determined by the powder particles’ melting state. Here, the printed layer’s width is defined by regime’s width (2*q*) where the powder elements are melted and can be calculated via the Gaussian distribution expression, as in ref. [29]:

(1) $$P\left(q\right)=\frac{2P}{\pi r_{l}^{2}}exp^{\left(\frac{-2q^{2}}{r_{l}^{2}}\right)}$$

Here, *P* is the laser power, and *r_l_* is the radius of the laser spot. The complete melting of powder particles, from ambient (*T_o_*) to melting temperature (*T_mp_*), requires the energy (*E_mp_*), calculated as [30]:

(2) $$E_{mp}=\frac{4}{3}\pi r_{p}^{3}\rho_{p}C_{p}^{*}.$$

In Equation (2), *r_p_* is the mean radius of powder particles, $\rho_{p}$ is the density of powder bed and $C_{p}^{*}$ is the modified specific heat value of the powder bed. The $\rho_{p}$ and $C_{p}$ values are calculated as ref. [31]:

(3) $$\rho_{p}=\left(1-\varepsilon \right)\rho_{b}.$$

(4) $$C_{p}=\left(1-\varepsilon \right)C_{b}.$$

Here,$\;\rho_{b}$, $C_{b}$ and ε are the density of the bulk material, the specific heat of the bulk material, and voids fraction in the powder bed, respectively. ε is calculated as:

(5) $$\varepsilon =1-\frac{\pi r_{p}^{2}}{S},$$

where *S* is the powder bed (PB) surface area, expressed as:

(6) $$S=\pi r_{pb}{}^{2}.$$

Here, *r_pb_* is the radius of the powder bed table. In Equation (2), $C_{p}^{*}$ is calculated as [30]:

(7) $$C_{p}^{*}=\frac{L_{fp}}{T_{mp}-T_{o}}+C_{p.}$$

Here, *L_fp_* is the enthalpy of fusion for a powder bed and $T_{o}$ is the room temperature. $T_{mp}$ is expressed as:

(8) $$T_{mp}=T\left(x,y\right)-T_{loss}.$$

T(x,y) is the laser-powder layer interaction temperature, while *T_loss_* is the temperature loss from the area where the laser beam heats the powder layer. T(x,y) is expressed as [31]:

(9) $$T\left(x,y\right)=T_{o}+\frac{P\alpha }{4\pi Rk}exp\left(\frac{V_{s}\;\left(R+x\right)}{2K_{p}\;}\right).$$

Here, *P* is the laser power, *V_s_* is the laser speed, *k* is the thermal conductivity of powder layer and substrate, *K_p_* (= *k*/*ρ_p_C_p_*) is the thermal diffusivity of the bulk substrate, α is the laser absorption coefficient of the powder particles and *R* is the distance from the laser beam to the substrate, expressed as:

(10) $$R=\sqrt{x^{2}+y^{2}+z^{2}}.$$

The correlation between laser power (*P*), laser intensity (*I*), and laser spot (*r_l_*) is expressed as [32]:

(11) $$P=\frac{I\pi r_{l}^{2}}{2}.$$

By substituting the above expression in Equation (9) will give:

(12) $$T\left(x,y\right)=T_{o}+\frac{Ir_{l}^{2}\alpha }{8Rk}exp\left(\frac{V_{s}\;\left(R+x\right)}{2K_{p}\;}\right).$$

The heat loss from the area where laser beam heats the powder (*Q*) is expressed as [33]:

(13) $$Q=UA\left(T\left(x,y\right)-T_{o}\right).$$

Here, *U* is the heat transfer coefficient of powder particles, and *A* is the area of the laser beam incident on the powder layer. *Q* can be converted from power to temperature as [34]:

(14) $$T_{loss}=\frac{Qt}{mH_{cap}}.$$

In Equation (14), *m* is the mass of the printed layer, *H_cap_* is the heat capacity of the powder layer and *t* is the laser-powder layer interaction time. Equations (15) and (16) give the analytical relations for *t* and *m* as [30]:

(15) $$t=\frac{2r_{l}}{V_{s}}.$$

(16) $$m=\frac{Q_{m}}{V_{s}}.$$

Here, *V_s_* is the laser scanning speed and *Q_m_* is the powder mass delivered by the powder re-coater (see Figure 1) and its analytical expression will be explained later. The energy balance can be applied on one of the powder particles as [30]:

(17) $$E_{mp}=\alpha \pi r_{p}^{2}t\frac{2P}{\pi r_{l}^{2}}exp^{\left(\frac{-2q^{2}}{r_{l}^{2}}\right)}.$$

As discussed above, the relation between the 1st printed layer’s width (*W*_1_) and position (*q*) where the powder particles are melted entirely, is expressed using the following expression [30]:

(18) $$W_{1}=2q.$$

The value of *q* can be calculated after comparing Equations (2) and (16), which is substituted in Equation (16) to calculate the expression for $W_{1}$ as:

(19) $$W_{1}=1.414r_{l}{\left[ln\left\{\frac{2r_{p}\rho_{p}C_{p}^{*}\pi r_{l}^{2}V_{s}}{3\alpha PD}\right\}\right]}^{\frac{1}{2}}.$$

It is worth mentioning that the thickness of the powder layer (*t_pl_*) is known and given as an input in the LPBF equipment. However, the laser melting and melt flow redefine the final thickness of the printed layer. In this study, the thickness of the layer has been assumed as the height of the printed layer. After estimating the 1st printed layer width, the law of mass conservation can be applied to calculate the 1st layer height (*h*_1_) as [30]:

(20) $$\forall_{2}Q_{m}t=\pi \frac{W_{1}}{2}\frac{h_{1}}{2}V_{s}t\rho_{p}.$$

In Equation (20), *Q_m_* is calculated as:

(21) $$Q_{m}=\rho_{p}V_{rc}t_{pl}l_{rc},$$

where *V_rc_*, $t_{pl}$ and *l_rc_* are the speed of re-coater, layer thickness, and length of re-coater responsible for delivering and spreading powder at the surface of the base plate (Figure 1), respectively. After solving Equations (20) and (21), one will achieve:

(22) $$h_{1}=\frac{4\forall_{2}\rho_{p}V_{rc}t_{pl}l_{rc}}{\pi W_{1}V_{s}\rho_{p}}.$$

To print the 1st layer, the laser energy arriving at the substrate’s depth (*Q_s_*) can be divided into two portions: (a) energy absorbed by the powder bed (*Q_p_*), and (b) energy used by the base-plate (*Q_BS_*) to generate melt-pool depth (*d*_1_), as [30]:

(23) $$Q_{S}=Q_{P}+Q_{BS}.$$

In Equation (23), $Q_{S}$ is calculated as:

(24) $$Q_{S}=\alpha \left(\frac{PL}{V_{s}}\right).$$

Here, *L* is the length of the printed layer. Furthermore, *Q_P_* can be expressed as:

(25) $$Q_{P}=\left(\forall_{2}\rho_{p}V_{rc}t_{pl}l_{rc}\frac{L}{V_{S}}\right)C_{p}^{*}.$$

Besides, *Q_BS_* has been calculated as [30]:

(26) $$Q_{BS}=\frac{\pi }{6}W_{1}d_{1}L\rho_{s}C_{s}^{*}.$$

In Equation (26), $C_{s}^{*}$ is the modified specific heat, which can be calculated using enthalpy fusion (*L_fs_*), temperature difference, and specific heat ($C_{s}$) of the substrate material as [30]:

(27) $$C_{s}^{*}=\frac{L_{fs}}{T_{ms}-T_{o}}+C_{s.}$$

Combining Equations (23)–(26) results in:

(28) $$d_{1}=\frac{\left[\forall_{2}\left(PL-V_{s}\right)\right]-\left[\forall_{2}\rho_{p}V_{rc}t_{pl}l_{rc}LC_{p}^{*}\right]}{\left[\frac{\pi }{6}\rho_{s}V_{s}W_{1}LC_{s}^{*}\right]}.$$

The printed layer’s width, height, and depth can be estimated using Equations (19), (22), and (28), respectively.

### 2.2. Multi-Layer Printing

**Figure 2:**
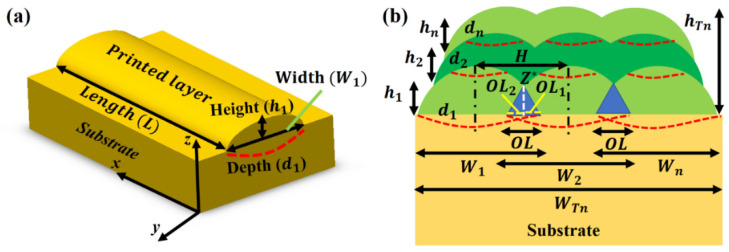
Schematic of laser powder bed fusion printing in the case of the (**a**) single-layer and (**b**) multi-layers.

For a given row, the depth of each printed layer is constant. Considering the 1st and 2nd rows, after depositing the layers in the 1st row, the laser beam generates a melt-pool depth in 1st layer to make bonding between 2nd and 1st layers. All printed layers have the same height and melt-pool depth for a given row. Figure 2a shows that 1st layer with the dimensions (*L* × *W*_1_ × *h*_1_) is printed with a melt-pool depth (*d*_1_) in the substrate (base plate). In LPBF, hatch distance (*H*) is essential for printing multiple layers, which defines the *OL* between two adjacent layers, shown by blue color in Figure 2b. This *OL* decreases the net width of the final manufactured part. To deposit the next row containing *n*-layers, the re-coater spreads a powder layer melted by the laser beam to a depth in the previously deposited layer to make a bonding. It reduces the net height of the manufactured component.

The 1st layer overlapping on the 2nd layer (*OL*_1_) can be calculated as:

(29) $$OL_{1}=\frac{W_{1}-H}{2}.$$

Similarly, the 2nd layer overlapping on the 1st layer (*OL*_2_) is expressed as:

(30) $$OL_{2}=\;\frac{W_{2}-H}{2}.$$

It is worthy of mentioning here that *W*_1_ and *W*_2_ are known as the operating conditions which are kept fixed. The total *OL* can be determined as:

(31) $$OL=OL_{1}+OL_{2}$$

Using Assumption 4, the area of *OL* can be calculated using the formula for an equilateral triangle area as:

(32) $$Area\;of\;OL=\frac{\sqrt{3}}{4}{\left(OL\right)}^{2}$$

The *OL* intersection (Z*) can be calculated as:

(33) $$Z^{*}=\frac{\sqrt{3}}{2}OL$$

The total width, after printing the two layers (*W_T_*_2_) in the 1st row, is expressed as:

(34) $$W_{T2}=W_{1}+W_{2}-\left(OL\right).$$

After estimating *W_T_*_2_, the *OL* (%) can be determined as:

(35) $$OL\;\left(\%\right)=\frac{OL}{W_{T2}}\times 100.$$

According to Assumption 3, all primary operating conditions are fixed, which results in the same height for the 2-printed layers, analytically, given as:

(36) $$h_{T1}=h_{1}.$$

The generalized relations for overlapping between any two adjacent printed layers are given as:

(37) $$OL_{n}=\frac{W_{n}-H}{2}.$$

(38) $$OL_{n+1}=\frac{W_{n+1}-H}{2}.$$

(39) $$OL=OL_{n}+OL_{n+1}.$$

The total width and height for *n*-number of layers are calculated as:

(40) $$W_{Tn}=\overset{n}{\underset{i=1}{\sum }}W_{i}-\overset{n}{\underset{i=1}{\sum }}OL_{i}.$$

(41) $$h_{Tn}=\overset{n}{\underset{i=1}{\sum }}h_{i}-\overset{n}{\underset{i=2}{\sum }}d_{i}.$$

In Equations (40) and (41), *n* (= 1, 2, 3…) is the number of layers.

### 2.3. Average Grain Dimension Estimation for Multi-Layers Printing

Within the 1st printed layer, the thermal stresses ($\sigma_{1th}$) can be calculated as [35]:

(42) $$\sigma_{1\left(th\right)}=\;\frac{E^{*}\beta }{1-v}\left[G\left(T_{1}\left(x,y\right)-T_{o}\right)\right],$$

where, *G*, *E*, β, and *v* are the material’s stiffness, elasticity modulus, thermal expansion coefficient, and Poisson’s ratio, respectively. For the 1st layer, the thermal strain rate ($\varepsilon_{o}$) is calculated as:

(43) $$\varepsilon_{1\left(o\right)}=\frac{\varepsilon_{1\left(th\right)}}{T_{1}\left(x,y\right)-T_{o}}.$$

Here, thermal strain ($\varepsilon_{th}$), can be determined as:

(44) $$\varepsilon_{1\left(th\right)}=\frac{\sigma_{1\left(th\right)}}{E^{*}},$$

where E* is the modified elasticity modulus. For metals, E* is expressed as [36]:

(45) $$E^{*}=E\frac{0.5T_{1}\left(x,y\right)}{T_{o}}.$$

In the LPBF process, the grain formation is primarily affected by the original grain dimensions, thermal intensity distribution, and strain rate due to temperature gradient. In this work, the Johnson–Mehl–Avrami–Kohnogorov (JMAK) model [37,38] is applied to compute the dynamic recrystallization (*X*_1*(drex)*_) in the 1st printed layer, expressed as [37,38]:

(46) $$X_{1\left(drex\right)}=1-exp^{-\left[0.693{\left(\varepsilon_{1\left(th\right)}-\frac{0.8\varepsilon_{1\left(p\right)}}{\varepsilon_{1\left(0.5\right)}}\right)}^{2}\right]}.$$

Here, *ε*_1*(p)*_ and *ε*_0.5_ are the peak strain and the strain at *X*_1*(drex)*_ = 0.5, and written as [37,38]:

(47) $$\varepsilon_{1\left(0.5\right)}=1.214\times 10^{-5}d_{o}^{0.13}\varepsilon_{1\left(o\right)}{}^{0.04}exp^{5.335\times 10^{4}/RT_{1}\left(x,y\right)}.$$

In Equation (47), $d_{o}$ is the initial grain size, which is equal to the mean powder particle diameter, *R* is gas constant (=8.3145 Jmol^−1^ K^−1^), and $\varepsilon_{1\left(p\right)}$ is expressed as [37,38]:

(48) $$\varepsilon_{1\left(p\right)}=4.107\times 10^{-3}\varepsilon_{1\left(o\right)}{}^{0.06}exp^{1.318\times 10^{4}/RT_{1}\left(x,y\right)}.$$

For 1st layer, the grain size ($d_{1\left(rex\right)}$), after dynamic recrystallization, is written as [37,38]:

(49) $$d_{1\left(rex\right)}=78.6022\varepsilon_{1\left(o\right)}{}^{-0.03722}exp^{-1902.72/RT_{1}\left(x,y\right)}.$$

After 1st layer printing, the average grain size ($d_{1\left(avg\right)}$) is calculated as [37,38]:

(50) $$d_{1\left(avg\right)}=d_{o}\left(1-X_{1\left(drex\right)}\right)+d_{1\left(rex\right)}X_{1\left(drex\right)}.$$

For 2nd layer, the dynamic recrystallization (*X*_2*(drex)*_) is calculated as:

(51) $$\sigma_{2\left(th\right)}=\frac{E^{*}\beta }{1-v}\left[G\left(T_{2}\left(x,y\right)-T_{o}\right)\right].$$

(52) $$\varepsilon_{2\left(o\right)}=\frac{\varepsilon_{2\left(th\right)}}{T_{2}\left(x,y\right)-T_{o}}.$$

(53) $$\varepsilon_{2\left(th\right)}=\frac{\sigma_{2\left(th\right)}}{E^{*}}.$$

(54) $$X_{2\left(drex\right)}=1-exp^{-\left[0.693{\left(\varepsilon_{2\left(th\right)}-\frac{0.8\varepsilon_{2\left(p\right)}}{\varepsilon_{2\left(0.5\right)}}\right)}^{2}\right]}.$$

(55) $$\varepsilon_{2\left(0.5\right)}=1.214\times 10^{-5}d_{o}^{0.13}\varepsilon_{2\left(o\right)}{}^{0.04}exp^{5.335\times 10^{4}/RT_{2}\left(x,y\right)}.$$

(56) $$d_{2\left(rex\right)}=78.6022\varepsilon_{2\left(o\right)}{}^{-0.03722}exp^{-1902.72/RT_{2}\left(x,y\right)}.$$

(57) $$d_{2\left(avg\right)}=d_{o}\left(1-X_{2\left(drex\right)}\right)+d_{2\left(rex\right)}X_{2\left(drex\right)}.$$

After depositing two layers, an average grain size ($d_{2L\left(avg\right)}$) is calculated as:

(58) $$d_{2L\left(avg\right)}=\frac{d_{1\left(avg\right)}+d_{2\left(avg\right)}}{2}.$$

For *n*-number of layers:

(59) $$\sigma_{n\left(th\right)}=\frac{E^{*}\beta }{1-v}\left[G\left(T_{n}\left(x,y\right)-T_{o}\right)\right].$$

(60) $$\varepsilon_{n\left(o\right)}=\frac{\varepsilon_{1\left(th\right)}}{T_{n}\left(x,y\right)-T_{o}}.$$

(61) $$\varepsilon_{n\left(th\right)}=\frac{\sigma_{n\left(th\right)}}{E^{*}}.$$

(62) $$X_{n\left(drex\right)}=1-exp^{-\left[0.693{\left(\varepsilon_{n\left(th\right)}-\frac{0.8\varepsilon_{n\left(p\right)}}{\varepsilon_{n\left(0.5\right)}}\right)}^{2}\right]}.$$

(63) $$\varepsilon_{n\left(0.5\right)}=1.214\times 10^{-5}d_{o}^{0.13}\varepsilon_{n\left(o\right)}{}^{0.04}exp^{5.335\times 10^{4}/RT_{n}\left(x,y\right)}.$$

(64) $$d_{n\left(rex\right)}=78.6022\varepsilon_{n\left(o\right)}{}^{-0.03722}exp^{-1902.72/RT_{n}\left(x,y\right)}.$$

(65) $$d_{n\left(avg\right)}=d_{o}\left(1-X_{n\left(drex\right)}\right)+\;d_{n\left(rex\right)}X_{n\left(drex\right)}.$$

After depositing *n*-number of layers, an average grain size ($d_{nL\left(avg\right)}$) is expressed as:

(66) $$d_{nL\left(avg\right)}=\overset{n}{\underset{i=1}{\sum }}\frac{d_{i\left(avg\right)}}{i}.$$

Here, *i* = 1, 2, 3, …, *n* (number of printed layers).

## 3. Material and Methods

**Figure 3:**
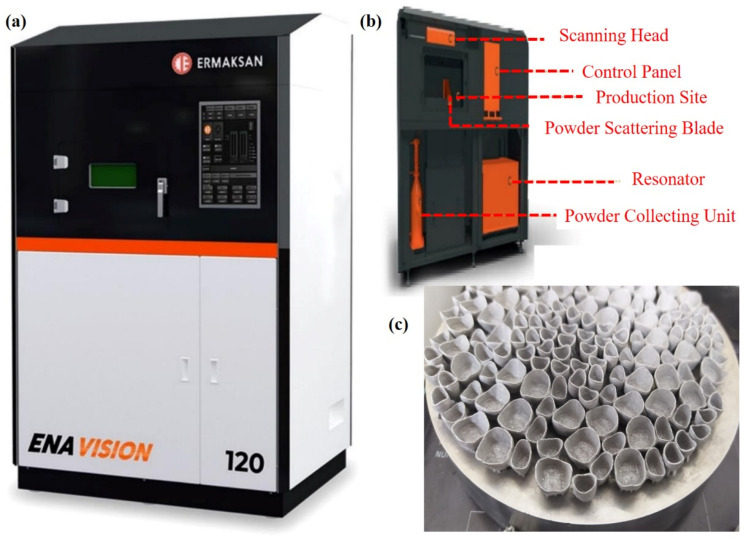
(**a**) ENAVISION 120 machine, (**b**) nomenclature of the machine, and (**c**) Co–Cr specimen printed by LPBF.

To validate the developed model, LPBF experiments were carried out using an ENAVISION 120 equipment (ERMAKSAN, Bursa, Turkey). This machine utilizes fiber laser and operates using maximum 300 W laser power and 11 m/s laser scanning speed. The focused laser spot size is 55 μm. For ENAVISION 120, further details can be obtained from Ref. [39]. Figure 3a shows the ENAVISION 120 machine utilized to validate the modelling results, Figure 3b provides the nomenclature of machine parts, and Figure 3c shows the Co–Cr specimens printed by LPBF.

For printing, Cobalt–chromium (Co–Cr) powder, made by ERMAKSAN, Turkey, was used in an 18–44 μm particle size range. All samples were prepared with 10 (length) × 10 (width) × 10 (height) mm^3^ dimensions; however, cylindrical supports having 2 mm dimensions were used for an easy removal of the printed samples. For experimentation, laser power (A1–A5), laser scanning speed (B1–B5), and hatch distance (C1–C5) were varied, while the powder layer thickness was kept constant. Table 1 compiles the operating conditions used to print Co–Cr samples.

**Figure 4:**
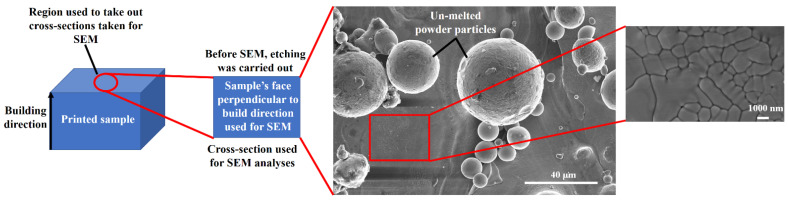
The methodology applied to conduct scanning electron microscopy (SEM) on the printed parts.

Figure 4 displays the methodology applied to determine the microstructure via SEM analysis. Before carrying out the SEM analyses, the cross-sections with 1 (length) × 1 (width) × 1 (thickness) mm^3^ dimensions were prepared for all the samples from the center of the printed cubes. All the samples were ground and polished, using SAPHI 520 (ATM, Mammelzen, Germany) equipment for microstructure analysis prior to the SEM analyses. For this purpose, Table 2 collects the steps applied for mirror-like polishing. All the samples were etched for 40 sec with HCL: HNO_3_ (3:1) solution as mentioned in Table 2. Later on, SEM was conducted at the prepared cross-sections, perpendicular to the build-direction. At 40 μm SEM magnification, several un-melted powder particles were analyzed. The same procedure was applied for all the deposited samples to correlate operating conditions and dendrite grain formation. It is worth mentioning that all the samples were printed and solidified at room temperature.

**Figure 5:**
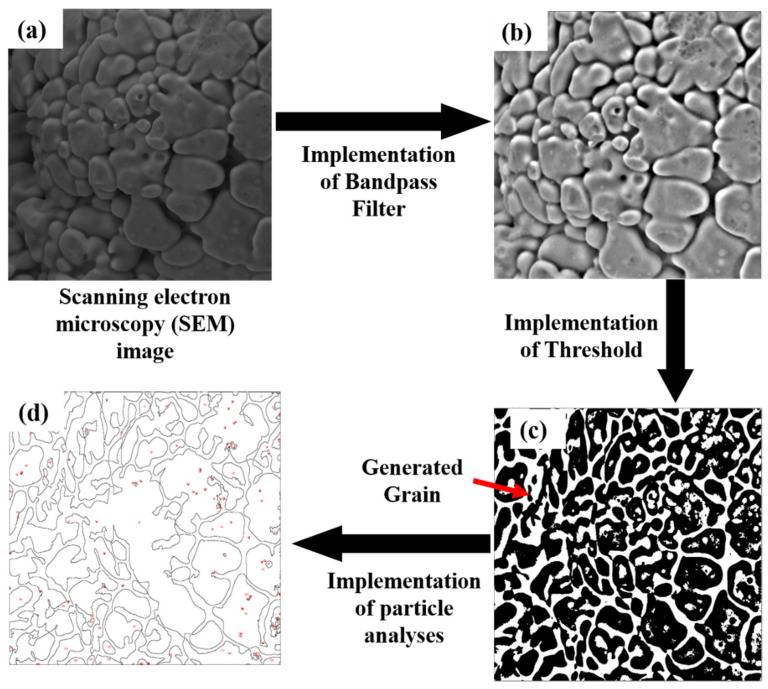
Implementation of “Image-J” software to calculate the average dendrite grain size in a SEM image (**a**) SEM image, (**b**) image after implementing bandpass filter, (**c**) image after implementing threshold filter, and (**d**) image achieved after particle analyses.

After preparation, the samples were examined with SEM by ApreoS instrument (Thermo Fisher Scientific, Waltham, MA, USA) and introduced in an image processing software “Image J” to quantify average grain size. The steps used in “ImageJ” software are provided in Figure 5a–d.

A Vickers hardness tester (FM-700, Future-Tech Corp, Tokyo, Japan) was used to test the hardness of the printed samples. A diamond-tip indenter was used. All items were polished to a mirror-like finish for testing, and the indentation was performed with a weight of 25 g.

## 4. Results and Discussion

**Figure 6:**
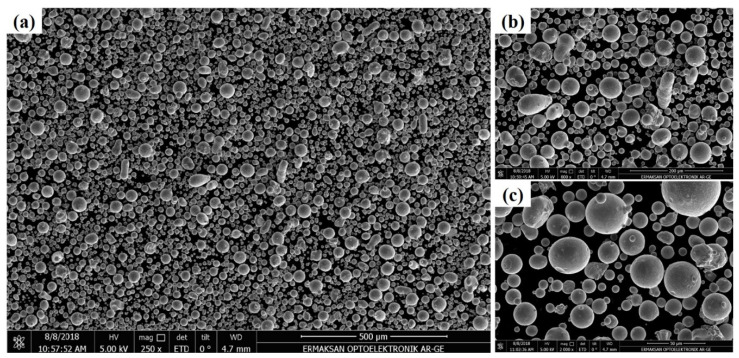
SEM images of Co–Cr powder particles (**a**) 500 µm, (**b**) 200 µm and (**c**) 50 µm.

Figure 6a–c shows the SEM images of Co–Cr powder particles at 500 µm, 200 µm, and 50 µm resolutions, respectively.

**Figure 7:**
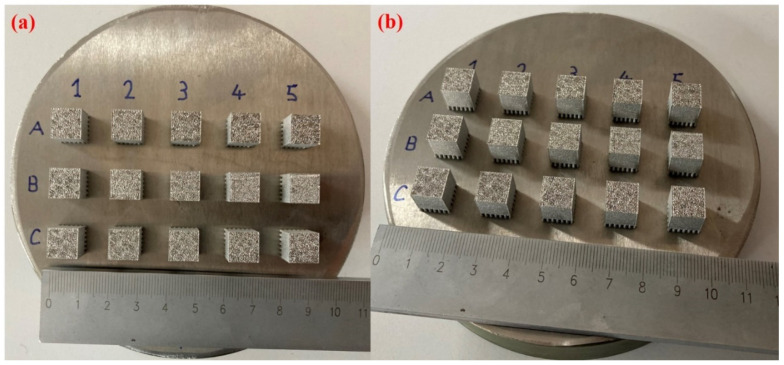
Fifteen samples printed using cylindrical supports by laser powder bed fusion process (**a**) top view and (**b**) inclined view.

Figure 7a,b present the 15 samples with dimensions 10 × 10 × 5 mm^3^, under different views, printed by LPBF. Cylindrical supports were used as the base of the samples, which helped an easy detachment of the workpieces from the substrate. On the other hand, a post-processing technique such as grinding is needed to smoothen the base region of the printed parts. From the images, one can observe the alphabetical classification as provided in Table 1.

**Figure 8:**
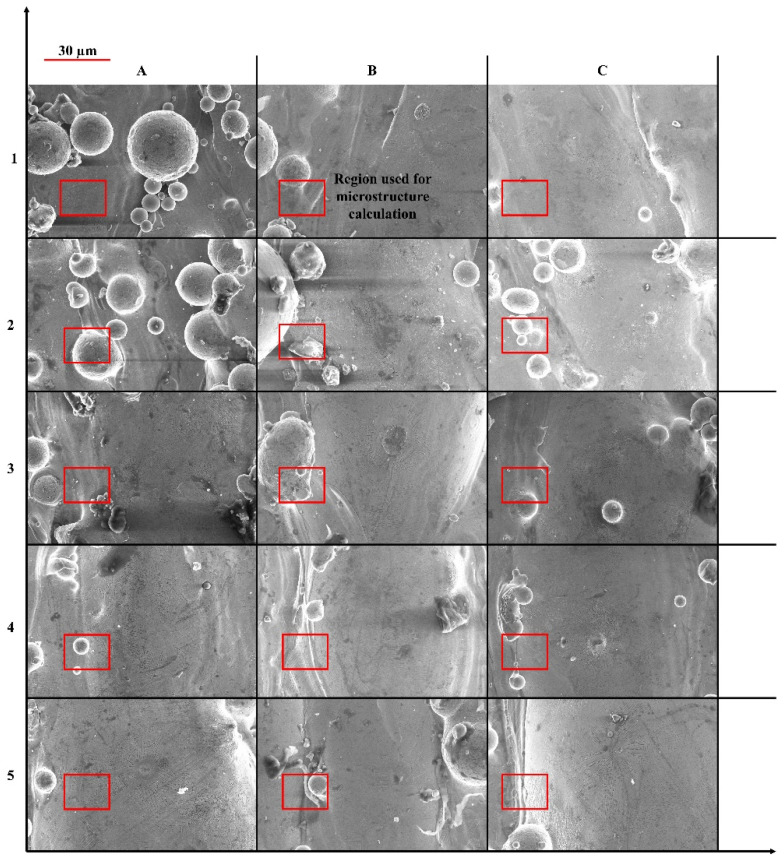
Morphologies of 15 printed samples with lack of fusion defects: A1–A5 (1st column), B1–B5 (2nd column), and C1–C5 (3rd column).

Figure 8 shows the morphologies of the 15 printed samples. A lack of fusion (LOF) defect has been identified in the printed samples, caused by the lack of energy input during the LPBF printing. In this type of defect, poor bonding and un-melted region are two primary reasons. A laser beam melts metallic powder particles selectively in the LPBF process. When the laser energy input is low, the diameter of the molten pool becomes small due to insufficient overlap between the tracks. Inadequate layer overlap between the scan tracks results in the un-melted regions formations. Remelting these powders becomes extremely difficult while adding a subsequent layer. As a result, the LPBF-fabricated part develops partial fusion holes. If the laser energy input is insufficient to induce a sufficient melt pool depth, LOF defects may occur due to insufficient interlayer bonding. Consequently, LOF defects in the scan tracks and deposited layers are frequently encountered. Additionally, the surface of a faulted area becomes rough, contributing to the molten metal’s inefficient flow, resulting in interlayer defects. Interlayer defects may gradually develop and propagate upward during a continuous deposition process for multi-layers printing [40,41]. From the figures, it can be analyzed that all the samples yield LOF-based defects. The areas marked with a red-colored square have been used for microstructure calculation utilized to validate the developed model and presented subsequently.

**Figure 9:**
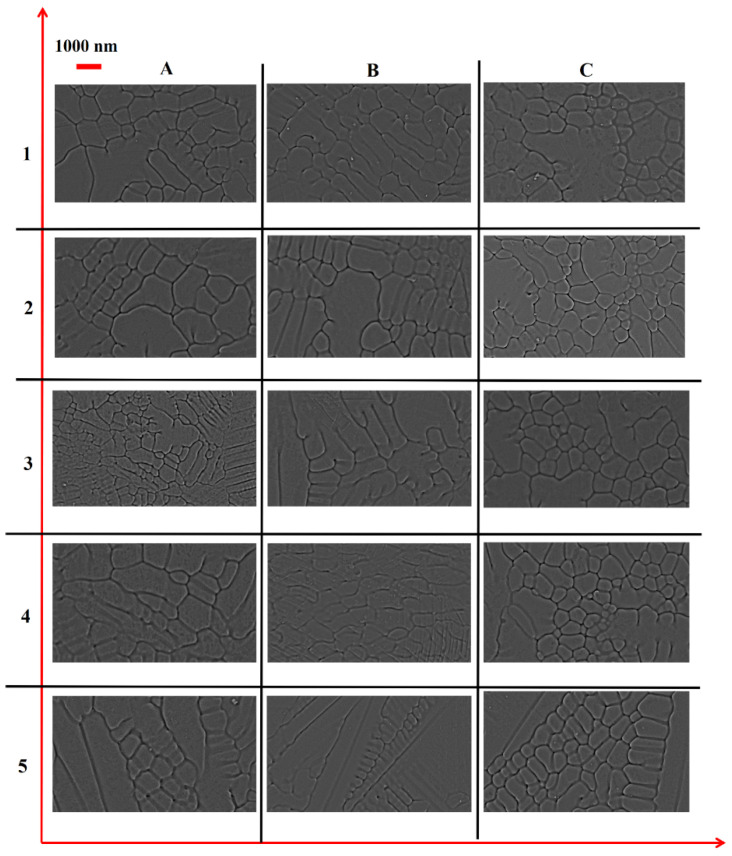
SEM images of A1–A5 (1st column), B1–B5 (2nd column), and C1–C5 (3rd column) samples.

Figure 9 shows SEM images of the 15 LPBF-ed samples. On the one hand, from Figure 9, it can be seen that a change in operating conditions only affects the dendrite grain dimensions and yields grains with a combination of two shapes: (a) near to circular and (b) elongated. On the other hand, the type of dendrite grain structure is a “single matrix phase of Co-based γ (FCC)” as identified based on ref. [42]. Kit et al. [42] carried out the x-ray diffractometry (XRD) of LPBF-ed Co–Cr alloys. It was found that the Co–Cr samples produced by LPBF presented the single matrix phase of Co-based γ-FCC-structures.

**Figure 10:**
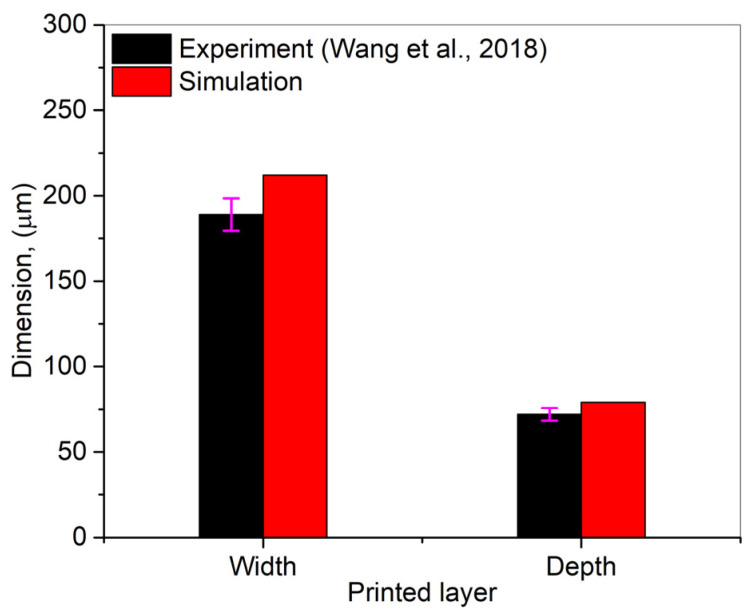
A comparison between experimental [43] and simulation width and depth of a single layer for Co–Cr alloy.

To validate the simulation model estimation for the printed layer’s width and depth, the authors have opted the published data from the study of Wan et al. [43]. In the mentioned study, the authors printed the samples with the following dimensions: 10 × 10 × 10 mm^3^ using Co–Cr alloy powder particles with laser power = 300 W, laser scanning speed = 900 mm/s, and hatch distance = 0.05 μm. The comparison results have been presented in Figure 10. It can be seen that the model was able to estimate results with 8–10% mean absolute deviations, which is due to the negligence of melt-flow surface tension during analytical modelling.

**Figure 11:**
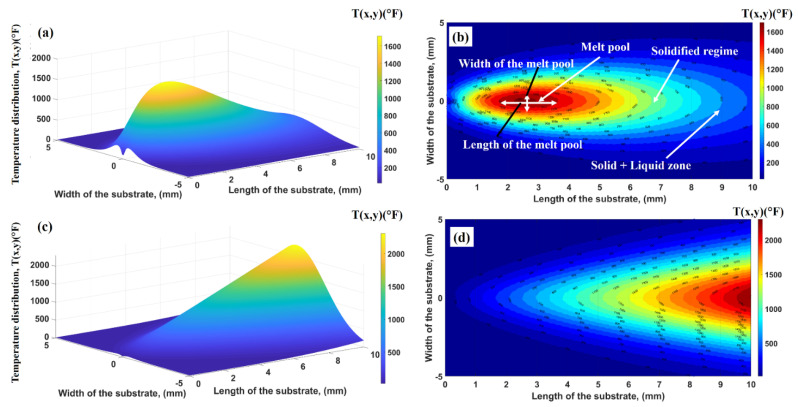
Three-dimensional and contour thermal distributions within the powder bed (**a**,**b**) when the laser beam is at the starting point and (**c**,**d**) when the laser beam arrives at the end of the substrate.

The thermal history plays an essential in defining the shape, grain size, and mechanical properties of [15]. In the LAM processes, the operating conditions are mainly responsible for estimating and controlling the thermal history within the deposited layers [16], which defines the grain formation. The thermal history with formed grains illustrates the deposited layers’ morphology and mechanical properties [18,44]. This phenomenon becomes complex with an increment in thermal cycles [45]. Figure 11a–c explains the computational results for laser power = 200 W and laser scanning speed = 1100 mm/s. A powder layer (dimensions: length = 10 mm, width = 10 mm, and height = 0.05 mm) is heated with a laser beam and allowed to translate along with the *x*-axis. In the case of LPBF, a powder layer has already been spread by the powder distributor whose length and width are equal to the length and width of the substrate. Figure 11a,b shows the 3D temperature and contour plots when the laser beam starts moving along the powder layer. The melt pool, mushy (solid + liquid) zone, and solidified regime are visible. It is important to mention that the melt pool characteristics play a critical role in defining the solidified region’s physical properties. In contrast, the mushy zone dynamics describe the evolution of microstructures and their distribution. Figure 11c,d illustrates the 3D thermal profile and contour plot as the laser beam reaches the end of the powder layer. It can be seen that as the laser beam is moving, the peak temperature value increases. This is because of the powder layer’s rapid heating and slow conduction. These phenomena are responsible for introducing the residual and thermal stresses in the printed material.

**Figure 12:**
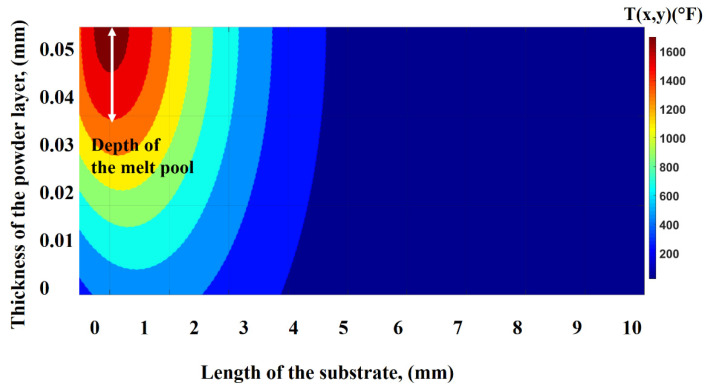
Thermal distributions across the thickness of the powder bed.

Figure 12 displays the temperature distribution across the depth of the powder layer. The red area shows the location of the laser beam at the top of the powder layer. The thermal isotherms travel from the top to the bottom of the layer, thus defining the melt-pool depth.

**Figure 13:**
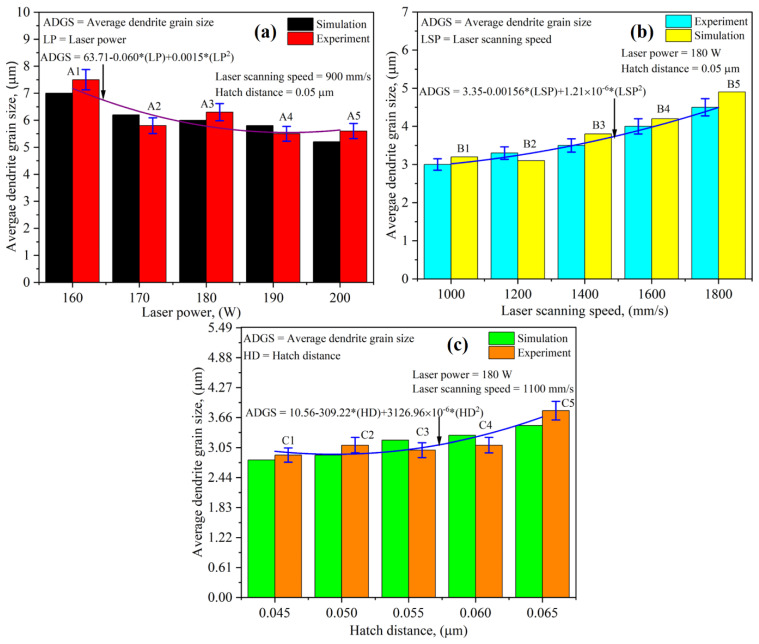
Effect of operating conditions on average dendrite grain size a correlation between experiments and simulations in the case of (**a**) laser power, (**b**) laser scanning speed, and (**c**) hatch distance.

Correlations between operating conditions and average dendrite grain size formation, including laser power, laser scanning speed, and hatch distance, were determined, as shown in Figure 13a–c. In the case of the laser power, it was found that its increment produces fine dendrite grains. This can be explained by the fact that when the laser power increases, it results in higher energy density at a given area, ultimately increasing the thermal gradient and solidification rate. This, in turn, offers dendrite grains with smaller dimensions [46]. A direct correlation has been found between laser scanning speed and average dendrite grain size for the laser scanning speed. When the laser beam travels with a low scanning speed, the thermal gradient and solidification rate decrease with the increment in volumetric energy density due to the higher amount of accumulated heat in the sample, yielding an elevated average dendrite grain size [46]. In the case of the hatch distance, the data trend shows that an increase in the hatch distance resulted in a higher average dendrite grain size value. A hatch distance is defined as the distance between two consecutive laser scans. Usually, this distance has an inverse correspondence with deposited layers overlapping. When this distance decreases, the previously deposited layer experiences cyclic thermal loading, thus reducing the average grain size after depositing the successive layer. This process becomes iterative when increasing the number of layers. Additionally, the experimental results were compared with the simulation results. From Figure 13, it can be observed that the computational results showed a deviation from experimental results of 10–15%. The results deduced using the current study presented better outcomes than those provided in ref. [24].

**Figure 14:**
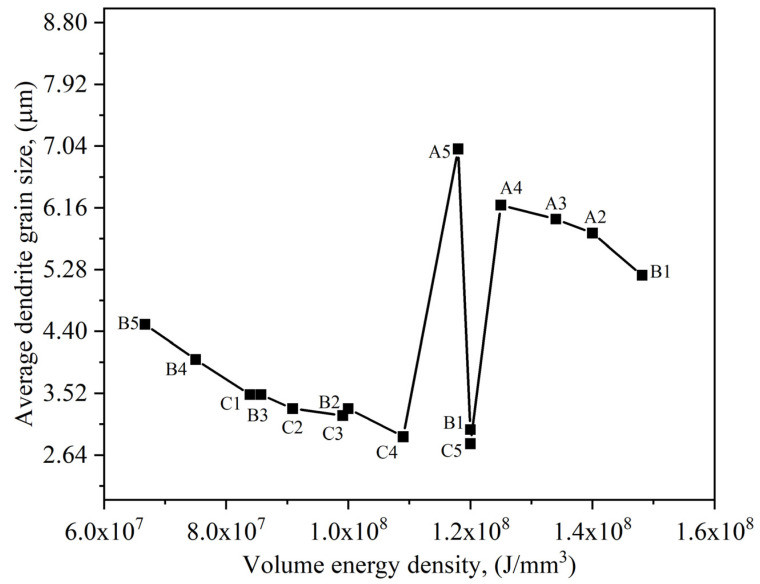
A correlation between volume energy density and average dendrite grain size.

In Figure 14, the dendrite grain sizes as a function of volumetric energy density are presented. From the results, one can identify two trends. One is for the sample series B and C, and another is for the sample series A only, suggesting that the solidification phenomena in sample series A, B, and C are completely different. Processing maps can be adapted to guide dendrite grain formation. Furthermore, thermal gradient (*G*) and solidification rate (*R*) can be translated into operating conditions to generate a process map easily interpreted. In the laser-based AM processes, *G* and *R* show a direct correlation [47]. A higher laser scanning speed causes an increment of *R*-value that promotes the formation of highly textured grains, while lower laser scanning speed favors equiaxed grains [47]. Conversely, increasing the laser power causes an increment in *G* [47]. It is important to mention that the *G* and *R* are not constant within the melt pool [47]. *R* is usually very high at the melt pool top and decreases linearly while moving from the top to the bottom [47].

**Figure 15:**
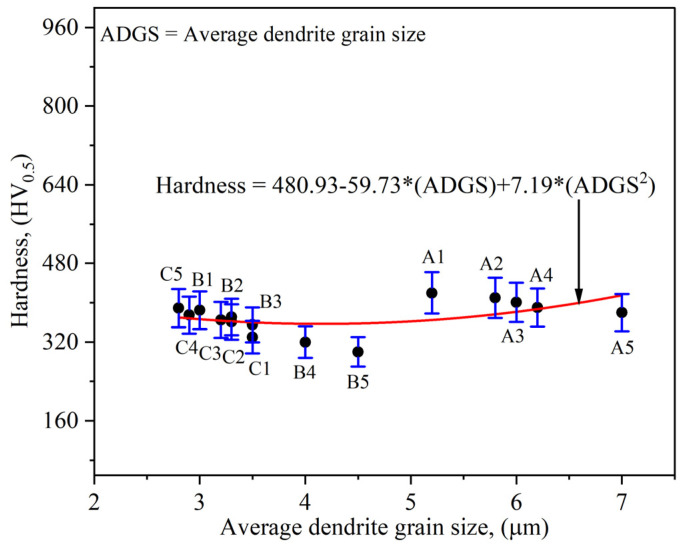
Relationship between average dendrite grain size and hardness value.

A relationship between the developed average grain size and corresponding experimental hardness value is shown in Figure 15. From the results, one can analyze that the average dendrite grain size is not affecting the hardness value significantly.

**Figure 16:**
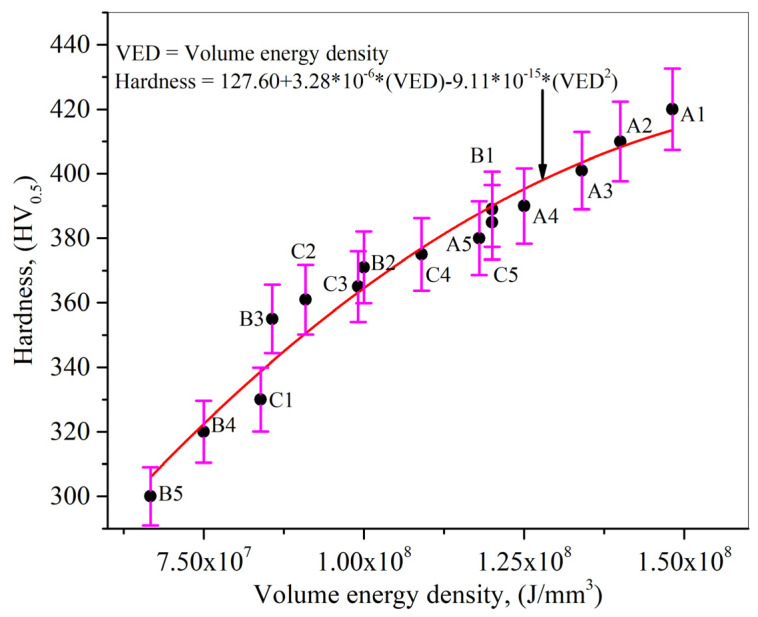
Correlation between volume energy density and Vickers hardness value.

Figure 16 displays the direct correlation between the laser volume energy density and hardness value. An increase in the laser volume energy density conveys more energy to a given area that causes more laser energy storage at that particular region. The indenter will need a higher load to enter that regime, resulting in an elevated hardness value. Upon comparing the results from Figure 15 and Figure 16, it can be concluded that the hardness value is defined by the volumetric energy, involving the thermal gradient and solidification rate, thus defining the printed samples’ morphology.

## 5. Conclusions

In this study, two analytical models have been developed for LPBF printing. Initially, the dimensions of a single printed layer have been estimated using primary operating conditions, including laser power, laser scanning speed, powder layer thickness, and hatch distance. The single-layer printing was converted into multi-layer printing using a hatch distance. The corresponding thermal history was used in the Johnson–Mehl–Avrami–Kolmogorov model to estimate the average dendrite grain size. LPBF experiments were carried out to validate the developed model for Co–Cr alloy printing. The developed model estimated results with a mean absolute deviation of 10–15%. The following conclusions have been developed based on the current study:

- Co–Cr LPBF-ed samples presented a single matrix phase of Co-based γ-FCC-structures. It was discovered that increasing the laser power resulted in smaller average dendrite grains for laser power. For LPBF simulation, melt pool, mushy zone, and the solidified regime were identified.
- When the laser power rises, the volumetric laser energy density increases, ultimately elevating the thermal gradient and solidification rate. Thus, yielding the dendrite grains with smaller dimensions. When the laser beam translates with a low scanning speed, the thermal gradient and solidification rate decrease with the increment in volumetric energy density, resulting in elevated average dendrite grain size. When the hatch distance decreases, the previously deposited layer experiences cyclic thermal loading, thus reducing the average dendrite grain size after depositing the successive layer.
- The laser volume energy density and hardness value have been discovered to have a direct relationship between them. It can be explained that when the laser volume energy density increases, more energy is delivered to a specific location, resulting in increased laser energy storage in that area. The indenter will need more energy to enter the specific region, resulting in a greater hardness value. In addition, the thermal gradient and solidification rate also control the printed samples’ morphology and, eventually, the final hardness value.

This study provides a time- and cost-efficient technique to identify the effect of operating conditions on average dendrite grain size. It will assist experimentalists in choosing operating conditions based on their specific requirements.

## Figures and Tables

**Table 1:** Operating conditions to print 15 specimens via the LPBF process.

Sample No.	Laser Power (W)	Laser Scanning Speed (mm/s)	Hatch Distance (µm)	Powder Layer Thickness (µm)
A1	200	900	0.05	0.03
A2	190	900	0.05	
A3	180	900	0.05	
A4	170	900	0.05	
A5	160	900	0.05	
B1	180	1000	0.05	
B2	180	1200	0.05	
B3	180	1400	0.05	
B4	180	1600	0.05	
B5	180	1800	0.05	
C1	180	1100	0.065	
C2	180	1100	0.06	
C3	180	1100	0.055	
C4	180	1100	0.05	
C5	180	1100	0.045	

**Table 2:** Step-by-step procedure to carry out grinding and polishing.

Name of Step.	Item Used for Processing	The Fluid Used for Sample Processing	Revolution/min	Applied Load (N)	Time
Grinding	Silicon carbide paper P320	Water	250	28	Until plane
Polishing	Alpha	Solution (9.0 µm with diamond)	150	24	5.0 min
	Gamma	Solution (3 µm with diamond)	150	24	5.0 min
	Lambda	Solution (0.06 µm with diamond)	150	18	2.0 min (water for 30 s at the end)
Etching	HCL:HNO_3_ (3:1)	-	-	-	40 s

## Data Availability

Not applicable.
